# Supplementary material for: Population coding of conditional probability distributions in dorsal premotor cortex
Source: Nat Commun. 2018 May 3;9:1788. doi: 10.1038/s41467-018-04062-6 (PMC5934453; doi:10.1038/s41467-018-04062-6)
Supplement: Supplementary file 1 — Supplementary Information [file 41467_2018_4062_MOESM1_ESM.pdf]

Population coding of conditional probability distributions in dorsal premotor cortex

Glaser et al.

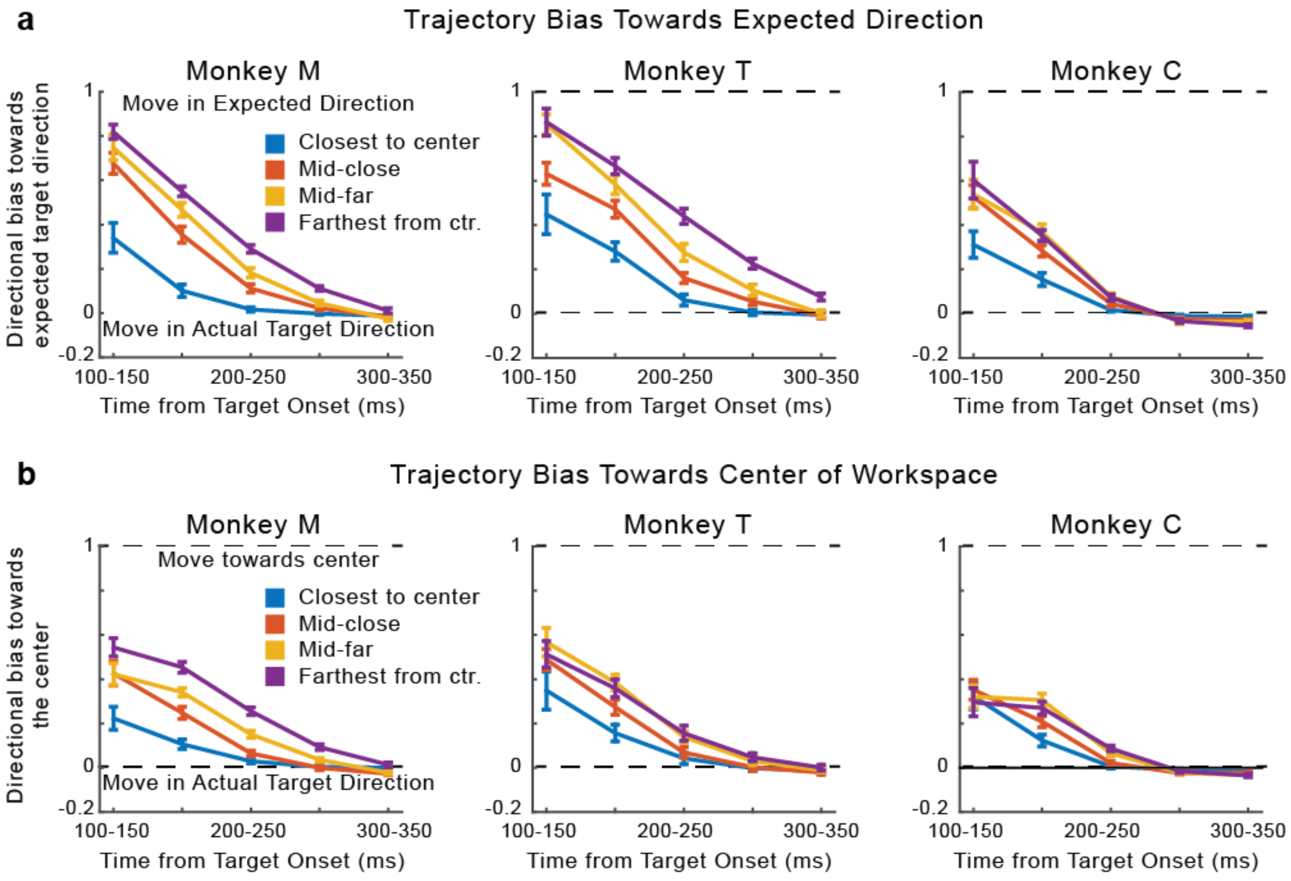

Supplementary Figure 1. Trajectory Biases

We compare **(a)** the biases in the reach direction towards the expected direction of the reach given the statistics of target presentation (identical to Fig. 2b) versus **(b)** the biases in the reach direction towards the center of the workspace.

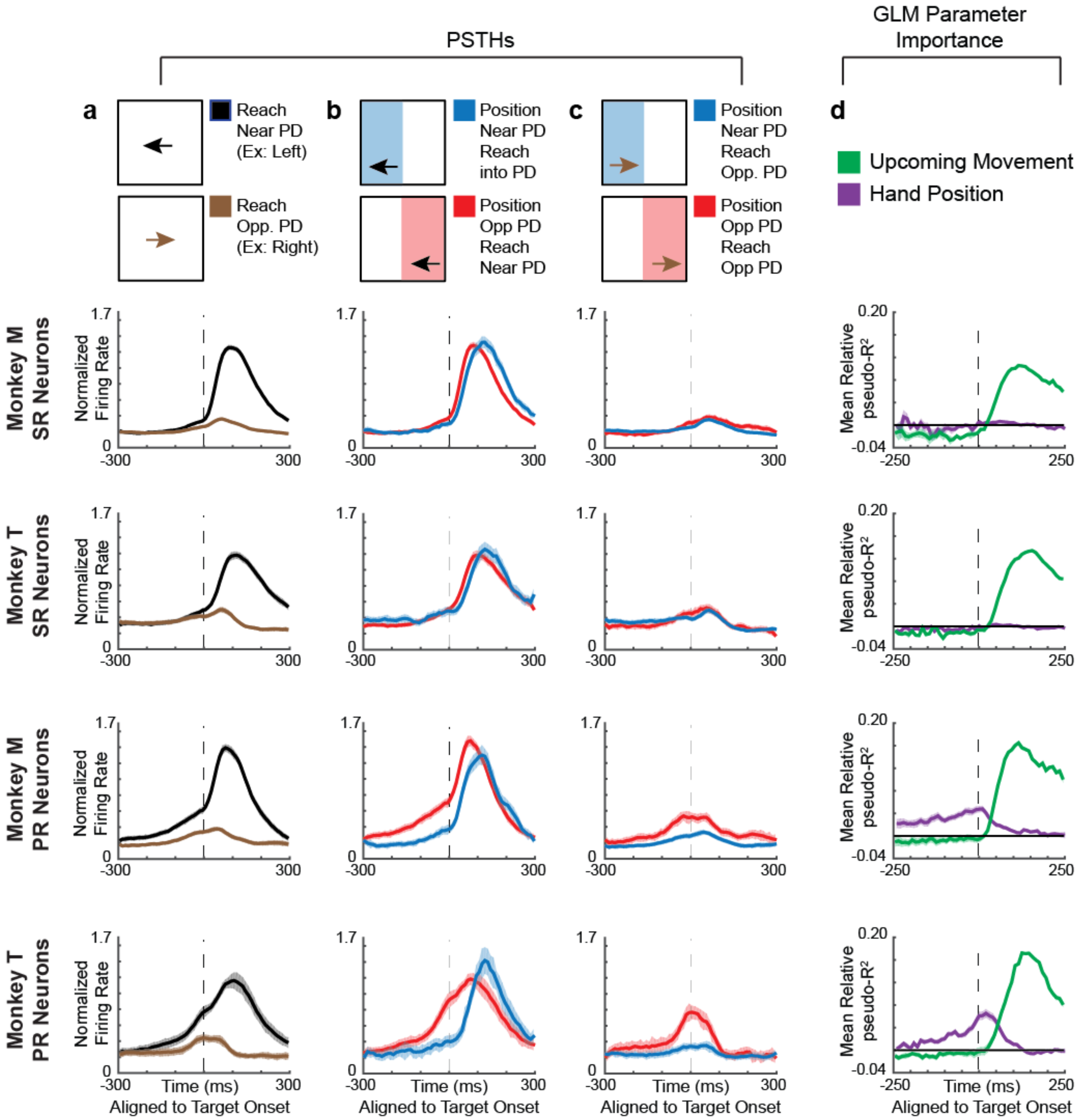

Supplementary Figure 2: PMd PSTHs and GLM results, for individual monkeys

Peristimulus time histograms (PSTHs) and GLM results for PMd neurons, for individual monkeys. All columns **(a-d)** are the same as in Fig. 3. **First and Second PSTH Rows**: Normalized averages of selected-response (SR) neurons from Monkey M and Monkey T, respectively. **Third and Bottom Rows**: Normalized averages of potential-response (PR) neurons from Monkey M and Monkey T, respectively.

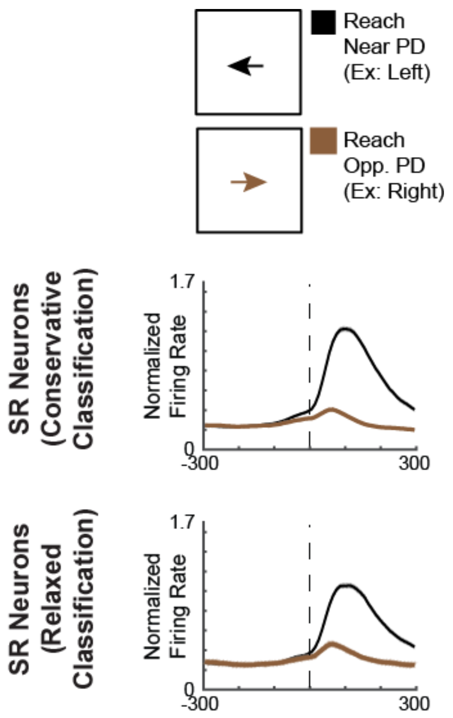

Supplementary Figure 3: Explaining pre-target activity for SR neurons

In Fig. 3, for SR neurons, prior to target onset there began to be a slight separation between activity traces depending whether the reach would be near vs. opposite the PD. Given that SR neurons are supposed to only respond after target onset, this is initially surprising. However, there are two likely reasons for SR neurons' apparent pre-target activity. The first reason is our classification criteria of SR and PR neurons. PR neurons, unlike SR neurons, were significantly modulated by hand position prior to target onset (see *Methods* for details). That is, if a neuron was modulated by hand position with > 95% (e.g 96%) confidence, then it was a PR neuron, but if it was modulated by hand position with < 95% (e.g. 94%) confidence, it would be an SR neuron. Thus, using this "conservative classification" (as we do in Fig. 3), we are likely including some PR neurons in the SR category. A PSTH using this conservative classification, copied from Fig. 3, is shown in the top row. Instead, if we use a "relaxed classification" that includes neurons as PR neurons if they are modulated by hand position with > 50% confidence, then SR neurons should not include any true PR neurons. When we plot SR neurons using this relaxed classification (bottom row), the differential activity prior to target onset diminishes, demonstrating that some PR neurons being included as SR neurons was a cause of the differential activity. Note that Supplementary Fig. 4 gives more details about different "conservative" and "relaxed" classification types. A second reason for the pre-target-onset differentiation of SR neurons in Fig. 3 is jitter in the time of target onset. While we subtracted the average delay for the target to be displayed on screen, there was some jitter in this timing (see *Methods*). Thus, some activity aligned to target onset could appear slightly earlier than it occurred.

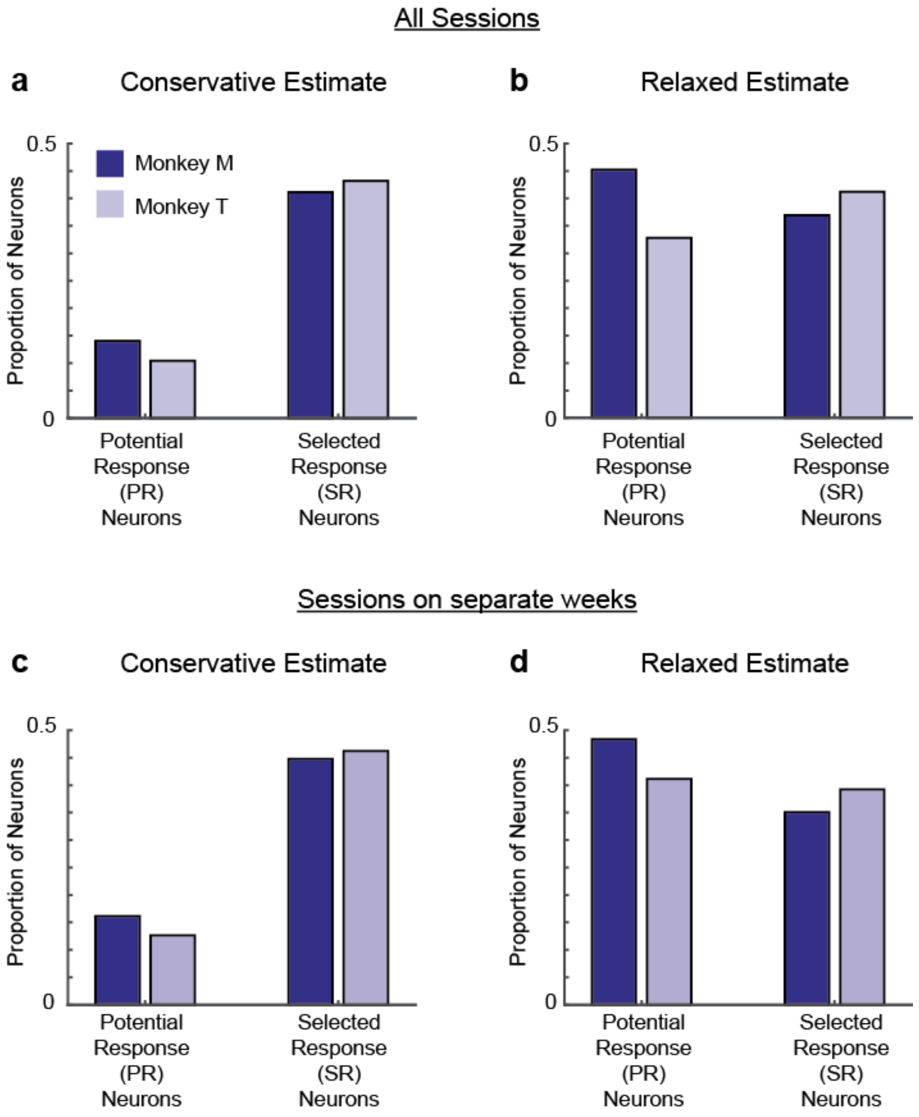

Supplementary Figure 4: Neuron Classification

The proportion of potential-response (PR) and selected-response (SR) neurons using different classification criteria. **a,b)** We include all sessions (as in the main text) **a)** We defined selected-response (SR) neurons as those that were significantly modulated by upcoming movement in the late period in the GLM, but were not significantly modulated by hand position in the early period. Potential-response (PR) neurons were significantly modulated by upcoming movement in the late period and by hand position in the early period. Significantly modulated means that the lower bounds of the 95% confidence intervals of pseudo-R^2 and relative pseudo-R^2 values were greater than 0 (see *Methods*). This was the criteria used for all parts of the main text with the exception of decoding. For monkey M, there were 73/520 PR neurons and 214/520 SR neurons. For monkey T, there were 26/250 PR neurons and 108/250 SR neurons. **b)** A more relaxed criteria is to look at all neurons that were significant at a level of 50% (median pseudo-R^2 and relative pseudo-R^2 > 0). Essentially, these neurons were on average modified by the covariates. In the main text, for decoding, we used this relaxed criteria to include PR neurons for decoding, in order to have a sufficient number of neurons. For monkey M, there were 235/520 PR neurons and 192/520 SR neurons. For monkey T, there were 82/250 PR neurons and 103/250 SR neurons. **c,d)** These are the same as panels a and b, respectively, except we now only include sessions that were recorded in separate weeks, to decrease the number of "repeat" neurons that were recorded in multiple sessions. **c)** Using the conservative classification criteria, for monkey M, there were 40/248 PR neurons and 111/248 SR neurons. For monkey T, there were 20/158 PR neurons and 73/158 SR neurons. **d)** Using the relaxed classification criteria, for monkey M, there were 120/248 PR neurons and 87/248 SR neurons. For monkey T, there were 65/158 PR neurons and 62/158 SR neurons.

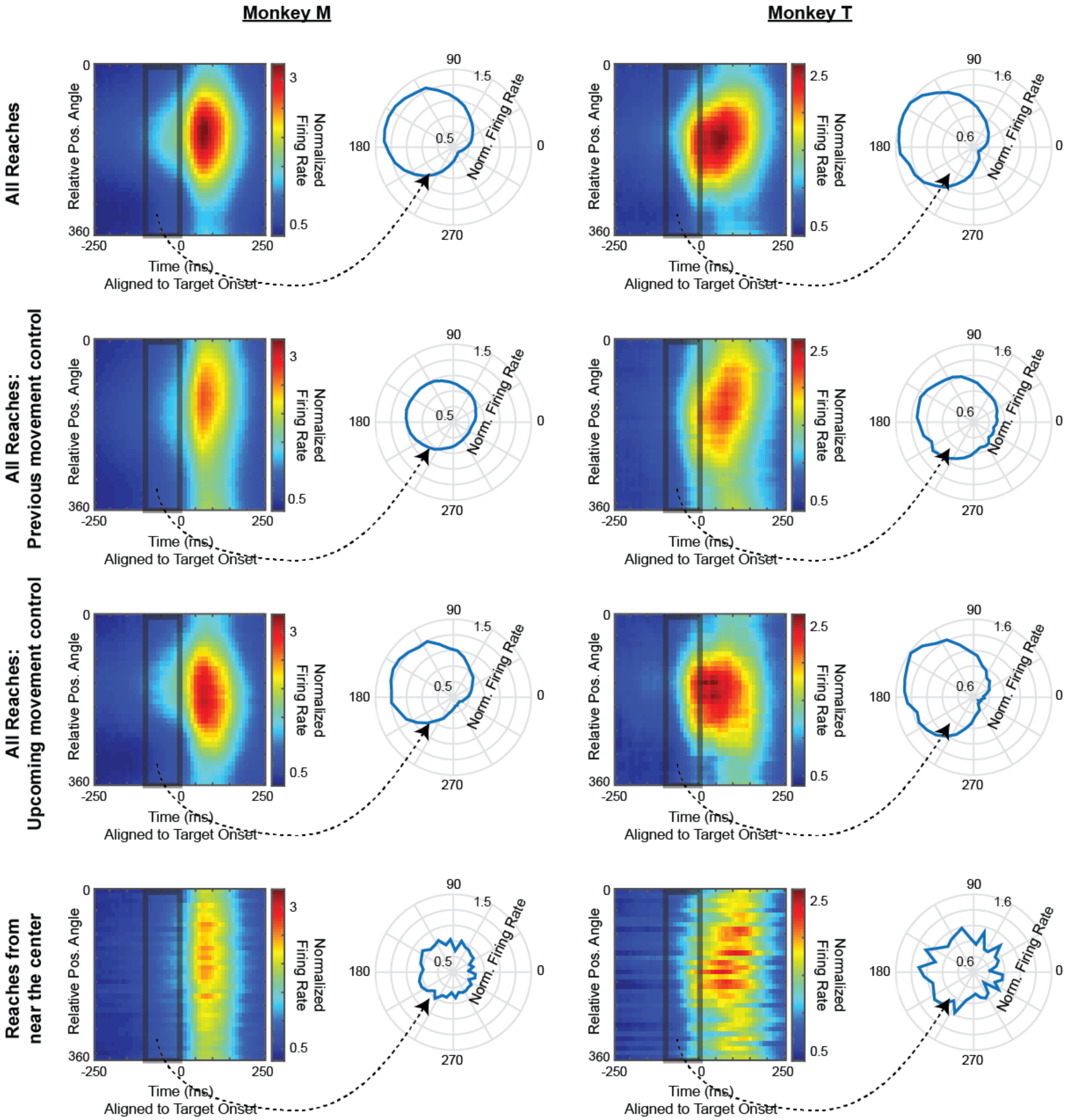

Supplementary Figure 5: PMd population activity represents the distribution of upcoming movements–accumulated across reaches: individual monkeys and controls.

As in Fig. 4a, all heat maps show normalized average activity over time as a function of relative angular position (the angular hand position relative to neurons' PDs). To the right of each heat map, normalized activity in the 100 ms prior to target onset is shown on a polar plot, as a function of the relative angular position. The **left** column is for monkey M, and the **right** column is for monkey T. Each row calculates activity from a different set of reaches. **Top row:** Normalized smoothed firing rate for all reaches (as in Fig. 4a). Note that both monkeys have peak angles of activity that are not significantly different from 150°. **Second row:** Normalized smoothed firing rate, controlling for the correlation between the previous movement direction and upcoming movement direction. One potential concern with the main results is that the neural activity might be related to the previous movement itself, rather than the probability distribution of upcoming movements. It is possible we were only capturing a correlation between the

previous and upcoming movements, which tended to be oppositely directed. As a control, we examined neural activity in the infrequent cases when pairs of sequential reaches were in similar directions (less than 90° away from each other). **Third row:** Normalized smoothed firing rate, controlling for the correlation between the angular hand position and upcoming movement direction. We resampled reaches to create a distribution of reach directions relative to angular hand position that was centered on 180° (rather than 150°). This plot ensures that our main results were not simply caused by a correlation with the true upcoming movement (in which case the activity after resampling would become centered on 180°), but rather reflected the distribution of upcoming movements (in which case the activity after resampling would remain centered at 150°). **Bottom row:** Normalized smoothed firing rate for reaches starting near the center (as in Fig. 4c).

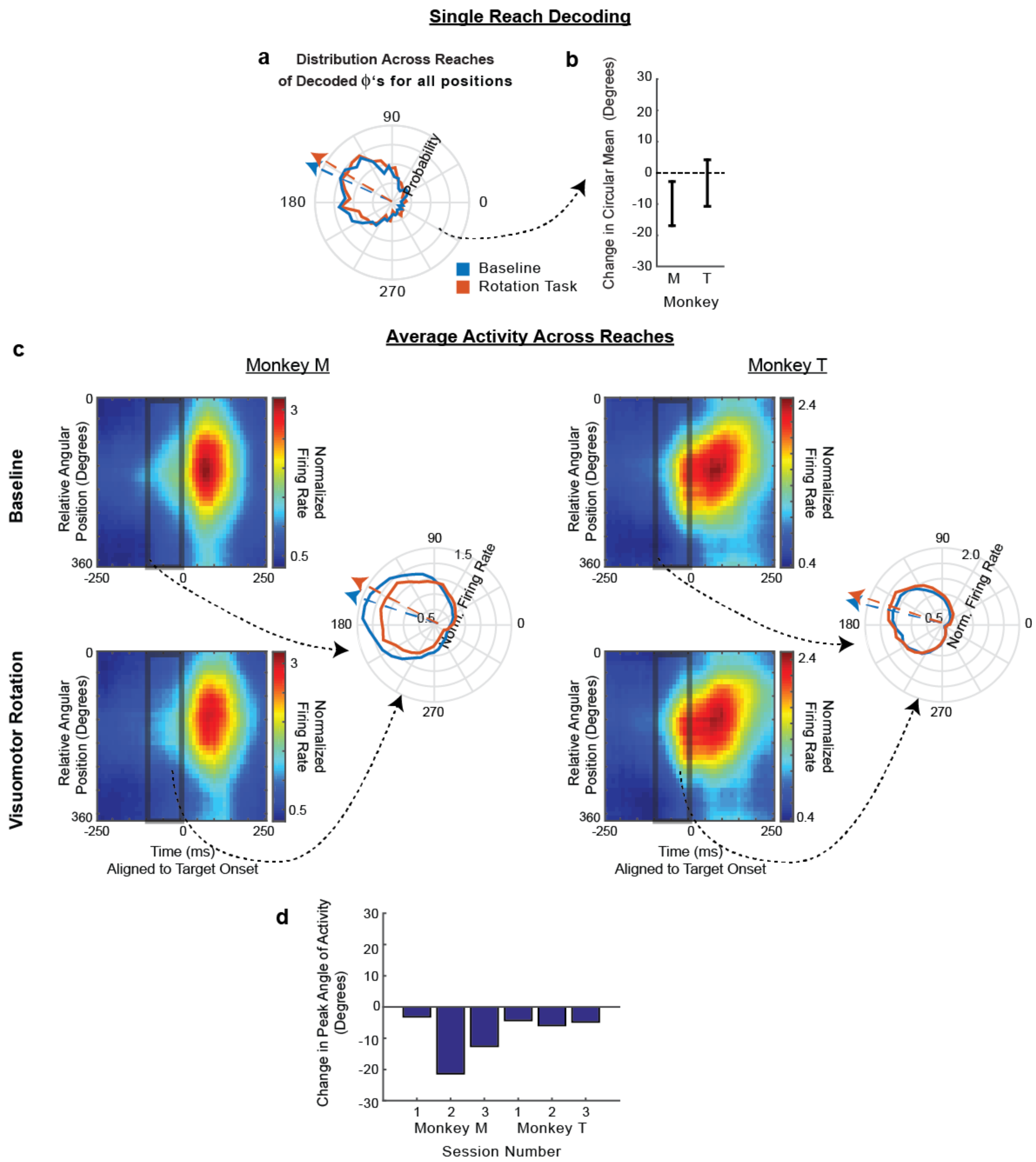

Supplementary Figure 6: Visuomotor Rotation Control Task

(a) The distribution of pre-target decoded reach directions relative to the hand's angular position (decoded Φ's) for all positions. Arrows point toward the circular means of the distributions. **(b)** The difference between the circular mean of the distributions of decoded Φ's in panel a, between the baseline and rotation tasks (rotation minus baseline). Error bars represent 95% confidence intervals from bootstrapping. **(c)** For each monkey, in both the baseline task and visuomotor rotation task, we display on the **left**: The average normalized firing rate of all PR neurons, over time, as a function of relative angular hand position. For each neuron, the relative angular position is the preferred direction of the neuron minus the angular hand position. Activity is normalized and averaged across all PR neurons. For each

monkey, we display on the **right:** The average normalized firing rate in the 100 ms prior to target onset, plotted as a function of the relative angular position (baseline in blue; rotation task in orange). Arrows point toward the peak angles of activity. **(d)** For each session, the difference between the angle corresponding to peak activity in panel c, between the baseline and rotation tasks (rotation minus baseline). In all panels, results from the VR task used the second 2/3 of trials.

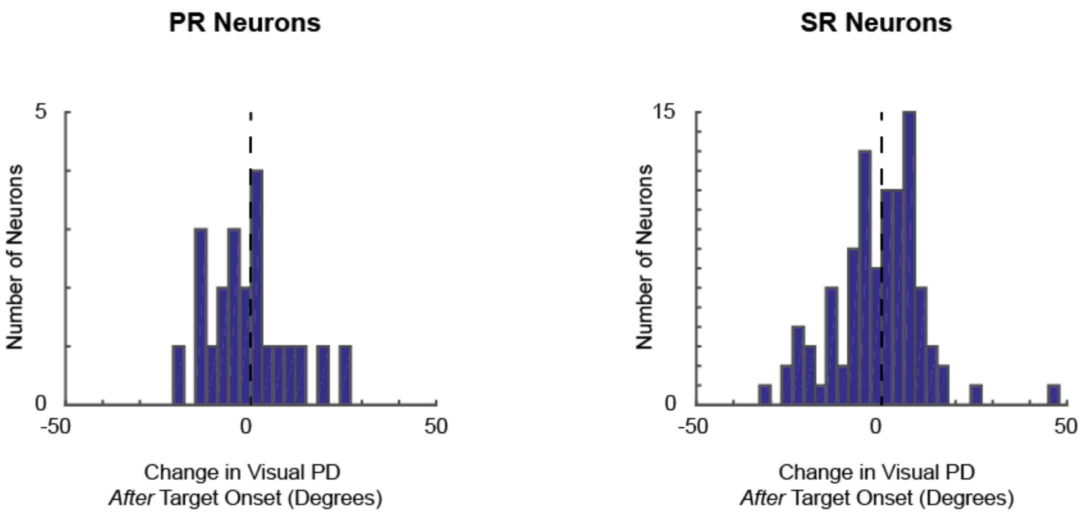

Supplementary Figure 7: Visuomotor Rotation Task – changes in PDs *after* target onset
On the **left**, we plot a histogram of changes in the visual PD for PR neurons, in the time period 50-200 ms after target onset. If the neurons were representing the movement rather than the target, then the neurons would be most active when the target is +30 degrees (counterclockwise) in the VR task. If they were representing the visual location of the target, then we would see a change of 0. The median change across PR neurons is -2.1 degrees. On the **right**, we have the same plot for SR neurons, with a median change of 0.4 degrees. Thus, the visuomotor rotation does not change PMd's representation of the target *after* target onset.

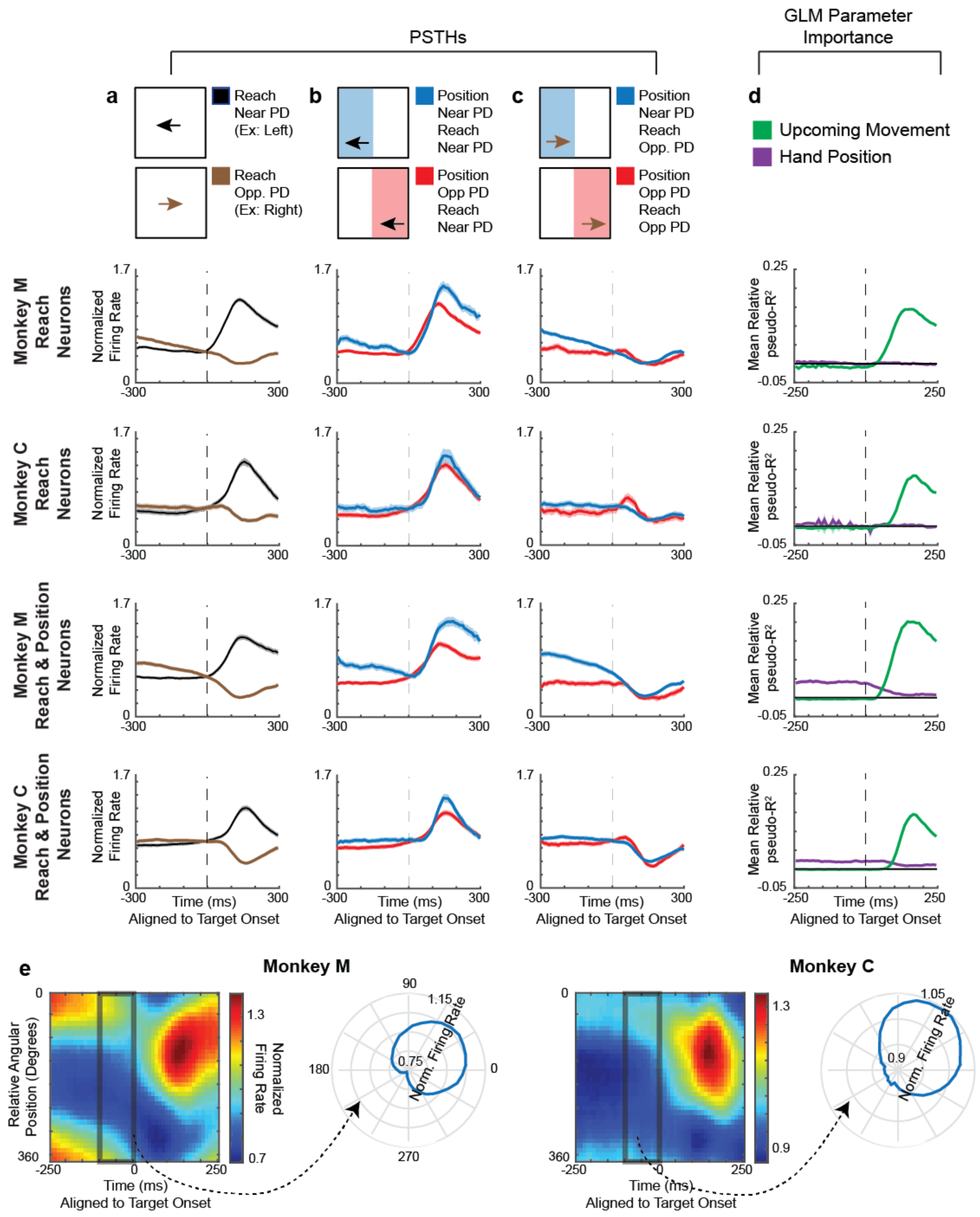

Supplementary Figure 8: M1 does not reflect the probability of upcoming movements, for individual monkeys (a-d) PSTHs and GLM results for M1 neurons. Columns have the same schematics as Fig. 3. **First and Second Rows of PSTHs**: Normalized averages of reach neurons, defined as those neurons significant for movement during the late

period, but not position in the early period of the GLM. **Third and Fourth Rows**: Normalized averages of reach & position neurons, defined as those neurons significant for movement during the late period, and position in the early period of the GLM. Note that we did not use the same "SR/PR" nomenclature as PMd, because there was not evidence in the PSTHs of M1 neurons that position was used to represent potential upcoming movements. **(e)** Same schematic as Fig. 4a, but for M1 neurons. **Left**: The normalized average firing rate, as a function of time and relative angular hand position. Activity is averaged across all reach & position neurons. **Right:** The normalized average firing rate in the 100 ms prior to target onset, plotted as a function of the relative angular position.

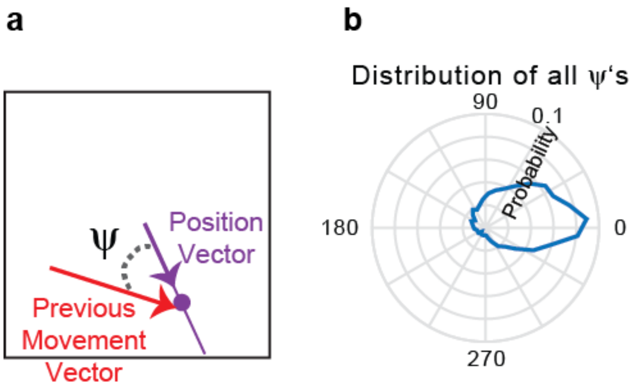

Supplemental Figure 9: Relationship between previous movement and hand position
(a) We define the angle between the previous movement vector and the hand position vector (relative to the center) as ψ. More specifically, ψ is the previous movement vector direction minus the position vector direction. **(b)** The distribution of ψ's, across all reaches. Note the slight counter-clockwise bias from 0 degrees (it is peaked at about 10° and has a circular mean at 23°), that may explain a portion of the counter-clockwise bias in M1 activity in Fig. 7e.
